# Toward a European definition for a drug shortage: a qualitative study

**DOI:** 10.3389/fphar.2015.00253

**Published:** 2015-10-30

**Authors:** Elfi De Weerdt, Steven Simoens, Minne Casteels, Isabelle Huys

**Affiliations:** Clinical Pharmacology and Pharmacotherapy, Department of Pharmaceutical and Pharmacological Sciences, KU LeuvenLeuven, Belgium

**Keywords:** drug shortages, supply problems, definition and concepts, semantics, qualitative research, literature review

## Abstract

**Background:** Drug shortages are currently on the rise. In-depth investigation of the problem is necessary, however, a variety of definitions for ‘drug shortages’ are formulated in legislations, by different organizations, authorities, and other initiatives. For international comparison, the underlying definition for drug shortages is important to allow appropriate interpretation of national databases and the results of scientific studies. The objective is to identify the different elements which should be considered in a uniform definition for drug shortages in the European Union (EU) and to detect the different conditions for reporting drug shortages.

**Materials and Methods**: Definitions of drug shortages were searched in the scientific databases as well as in the gray literature. Similar topics were identified and organizations were contacted to formulate the reasoning underlying the definitions.

**Results**: Over 20 different definitions for drug shortages were identified. A distinction is made between general definitions of drug shortages and definitions used for the reporting of drug shortages. Differences and similarities are observed in the elements within the definitions, e.g., when does a supply problem become a drug shortage, permanent and/or temporally shortages, the typology and time frame of a drug shortage. The moment a supply problem is considered as a shortage, can be defined at four levels: (i) demand side, (ii) supply side, (iii) delivery of a drug, and (iv) availability of a drug. Permanent discontinuations of drugs are not always covered in definitions for drug shortages. Some definitions only consider those drugs used for the treatment of serious diseases or drugs for which no alternative is available. Different time frames were observed, varying between 1 day and 20 days.

**Conclusion**: Obtaining a uniform definition for drug shortages is important as well as identifying which conditions are preferable to report drug shortages in order to facilitate international benchmarking. This paper can be used as a guidance to point out all the different elements which should be considered to formulate a uniform definition to be applied in the EU.

## Introduction

Recently, drug shortages have become a common problem in European Union (EU) Member States (European Association of Hospital Pharmacists, 2014). During the past decades, the awareness for drug shortages has been grown due to the observation of an increased number of drug shortages. Different stakeholders are engaged in the problem. In the first place, hospital as well as community pharmacies are affected by drug shortages. Patients are also troubled by this problem as well as medical doctors and nurses. They might be less familiar with the alternative treatment in case of a drug shortage, which can lead to medication errors (Pharmaceutical Group of the European Union, 2012; Yurukoglu, 2012; European Association of Hospital Pharmacists, 2014; Heiskanen et al., 2014). Different umbrella organizations of the pharmaceutical industry published papers on drug shortages, some of them including guidelines on how to prevent and reduce risks for shortages. These actions suggest that drug shortages are also to the detriment of the pharmaceutical companies, due to the reputational damage it may provoke (European Association of Pharmaceutical Full-line Wholesalers (GIRP), 2013; European Federation of Pharmaceutical Industries and Associations, 2013; ISPE, 2014). National competent authorities are also burdened with the problem of drug shortages. Due to the EU Directive 2001/83/EC, national competent authorities should control the continue supply of drugs and should be warned by the suppliers of every cessation of pharmaceutical products whether this is temporally or permanently (The European Parliament and the Council of the European Union, 2011; De Weerdt et al., 2015).

Until recently, studies on drug shortages were mainly found in US literature (Kaakeh et al., 2011; Ventola, 2011; Kweder and Dill, 2013; Woodcock and Wosinska, 2013; Fox et al., 2014). These studies investigated the scope, the causes and impact of drug shortages in the US. In recent years, more research concerning drug shortages in Europe has been published, trying to gage the scope and causes of drug shortages (Costelloe et al., 2014; Heiskanen et al., 2014; Pauwels et al., 2014, 2015). However, the results of these studies are hard to compare as different definitions of drug shortages have been used.

Should a drug supply problem always be considered as a drug shortage or should it only be considered when patients have no longer access to a particular medicine? Are drug shortages taken into account when drugs cannot be delivered for only one day and thus should a time frame be considered in the definition of drug shortages? When manufacturers retract a product from the market, should it be considered as a drug shortage?

The objective of this paper is to identify the essential elements which are crucial in a definition for drug shortages and to identify which conditions are preferable to be considered when reporting drug shortages in databases. In order to achieve these aims, it was important to understand the reasons why certain organizations decided to develop their own drug shortage definition.

A uniform definition at EU level together with its reporting conditions will facilitate the problem of drug shortages at different levels. Firstly, international comparison will be facilitated, especially if the national databases of the reported drug shortages are publicly accessible, e.g., national competent authorities can easily control whether a drug shortage is an international or national problem. This information can also help them to find potential solutions. Secondly, the scope of drug shortages can be objectively estimated. Understanding the scale of the problem will help to take appropriate action against drug shortages by industry as well as national authorities. And thirdly, a uniform definition will also facilitate the communication between different stakeholders.

## Materials and Methods

Because the nature of the study, it was not required to seek approval from a research ethics committee. Data were analyzed anonymously.

Data were collected in the scientific literature as well as in the gray literature between October the 6th 2014 and April the 31th 2015. Scientific articles were identified in the following databases: MEDLINE, Embase, and Web of Science. The search strategy was developed incorporating synonyms for “drug shortages” combined with “defin*”. By using an asterisk every word starting with “defin” was included. Individual search terms and MeSH search terms were used alone and in combinations. Studies published in English, French, Dutch, and German were included in the paper. Additionally, the bibliography of the retrieved papers was checked for other relevant articles or studies. Gray literature, which are websites and documents of different stakeholders, was also searched for definitions of drug shortages. Some examples of possible sources which were searched for definitions are: legislations (e.g., Belgian law, EU Directives, etc.); governmental organizations (e.g., AIFA etc), professional organizations [FIP; European Hospital Pharmacists Associations (EAHP), etc.]; patient organizations (e.g., European Public Health Alliance, etc.). The same language criteria as described above were applied to gray literature documents.

Definitions were analyzed according to their literary differences and similarities. Main topics as: “the level of supply,” “permanent or temporally discontinuation,” “time limit,” and “typology” were identified. These topics were investigated and observed differences and similarities were subdivided.

To identify the reasoning behind the wordings within the identified general definitions, the organizations and national authorities that formulated those definitions were contacted. Three main questions were asked: (i) “when should supply problems be considered as drug shortages? Should drug shortages include supply problems when patients are not affected (meaning: when patients have to switch to another (generic) medicine, they are affected)?”; (ii) “do you believe a time frame is important in the definition of a drug shortage? Please reason why”; and (iii) “Does the definition also include drugs which have been permanently discontinued? Please explain”. These questions were sent via email to different organizations (seven national and four international). Answers were obtained from four national organizations and two international organizations. Appropriate quotes were selected from the answers and were translated from Dutch to English. Deleted parts of the quotes are indicated by “…”. The coding [**R1, R2, etc.**] identifies the respondent.

## Results

From the literature review 26 different definitions for drug shortages are identified (Wet Betreffende de Verplichte Verzekering Voor Geneeskundige Verzorging En Uitkeringen Gecoördineerd Op 14 Juli, 1994; Fox et al., 2009; Food and Drug Administration, 2011a; Dragic, 2012; Executive Agency for Health and Consumers, 2012; Instituut voor Verantwoord Medicijngebruik, 2012; Charnay-Sonnek et al., 2013; European Federation of Pharmaceutical Industries and Associations, 2013; European Medicines Agency, 2013; Fédération Internationale Pharmaceutique, 2013; ISPE, 2013; ANSM, 2014; Costelloe et al., 2014; Government of Canada - Health Canada, 2014; Federaal Agentschap voor Geneesmiddelen en Gezondheidsproducten, 2014; Heiskanen et al., 2014; Koninklijke Nederlandse Maatschappij ter bevordering der Pharmacie, 2014; The Italien Medicines Agency, 2014; Agencia Española de Medicamentos Y Productos Sanitarios - AEMPS, 2015; Australian Government - Department of Health - Therapeutic Goods Administration [TGA], 2015; BfArM - Lieferengpässe, 2015; Canadian Drug Shortage Database, 2015; Décret N° 2012-1096 Du 28 Septembre 2012 Relatif À L’approvisionnement En Médicaments À Usage Humain, 2015; Pauwels et al., 2015). Different sources are acknowledged, including: national laws, governmental and professional organizations, and scientific articles. All definitions found are unique, with the exception that most scientific articles refer to already existing definitions of the ASHP and/or the FDA (Balkhi et al., 2013; Becker et al., 2013; Wiggins et al., 2014). The definitions will be divided into three categories: (i) general definitions for drug or medicine shortages, (ii) definitions specifying the conditions to report drug shortages, and (iii) definitions found in articles. It occurs that in some countries more than one definition is exploited: e.g., a definition for a drug shortage (national law) and a definition for the purpose of reporting a drug shortage (federal agency) (Wet Betreffende de Verplichte Verzekering Voor Geneeskundige Verzorging En Uitkeringen Gecoördineerd Op 14 Juli, 1994; Fox et al., 2009; Food and Drug Administration, 2011b; Federaal Agentschap voor Geneesmiddelen en Gezondheidsproducten, 2014).

The four definitions of drug shortages found in scientific articles will not be discussed in further detail, because the aim of these definitions is to clarify which shortages are covered by the scope of such paper.

In total 14 general definitions for drug shortages are found, including two references of national laws (Belgium and France), five of governmental organizations, six papers of professional organizations and a definition of the EMA which was formulated in 2014, however, at the current time, that definition is removed from the EMA website (Wet Betreffende de Verplichte Verzekering Voor Geneeskundige Verzorging En Uitkeringen Gecoördineerd Op 14 Juli, 1994; Fox et al., 2009; Food and Drug Administration, 2011b; Executive Agency for Health and Consumers, 2012; Instituut voor Verantwoord Medicijngebruik, 2012; Charnay-Sonnek et al., 2013; European Federation of Pharmaceutical Industries and Associations, 2013; Fédération Internationale Pharmaceutique, 2013; ISPE, 2013; Government of Canada - Health Canada, 2014; The Italien Medicines Agency, 2014; Australian Government - Department of Health - Therapeutic Goods Administration [TGA], 2015; Décret N° 2012-1096 Du 28 Septembre 2012 Relatif À L’approvisionnement En Médicaments À Usage Humain, 2015). An overview of these definitions can be found in **Table 1**.

**Table 1:** Overview of the general definitions of drug shortages.

Source	Definition
**Legislations**
Belgian law (Wet Betreffende de Verplichte Verzekering Voor Geneeskundige Verzorging En Uitkeringen Gecoördineerd Op 14 Juli, 1994) On compulsory insurances for medical care coordinated on 14th July 1994 – art. 72 bis	A drug is unavailable when enterprises which are responsible for the marketing of the drug are unable to deliver that drug for an uninterrupted period of four days to the community pharmacies, hospital pharmacies, or wholesalers in Belgium.
French law (Décret N° 2012-1096 Du 28 Septembre 2012 Relatif À L’approvisionnement En Médicaments À Usage Humain, 2015) Decree n° 2012-1096 the 28th of September 2012 on the supply of medicines for human use	A supply disruption is the inability for a dispensary pharmacy or internal use pharmacy dispensing a drug to a patient within 72 h or within a shorter time depending on the compatibility problems with the continued treatment of the patient.
**Governmental organizations**
Dutch Institute for Rational Use of Medicine (Instituut voor Verantwoord Medicijngebruik, 2012)	The (temporally) not or inadequate supply of a registered pharmaceutical.
AIFA (The Italien Medicines Agency, 2014)	When a medicinal product is not available or commercially unavailable all over the country and the market authorization holder does not assure appropriate and continued supply to meet the patients’ needs.
EMA	When the delivery of a medicine cannot comply to the needs of the patients, whether this is local, national, or international.
FDA (Food and Drug Administration, 2011b)	A situation in which the total supply of all clinical interchangeable versions of a FDA-regulated drug is inadequate to meet the current or projected demand at the patient level.
Health Canada (Government of Canada - Health Canada, 2014)	A drug shortage is a situation when a manufacturer or importer of a drug cannot meet actual or projected demand. Drug shortages can include temporary disruptions or permanent discontinuances in the production and supply of a drug.
Australian Ministry of Health (Australian Government - Department of Health - Therapeutic Goods Administration [TGA], 2015	A medicine shortage occurs when the supply of a medicine is not likely to meet the normal or projected consumer demand for the medicine within Australia for a period of time.
**Professional organizations**
ISPE (ISPE, 2013)	A situation in which the total supply of an approved (by the appropriate Health Authority) drug is inadequate to meet the current or projected demand at the user level.
EFPIA (European Federation of Pharmaceutical Industries and Associations, 2013)	A potential drug shortage is defined as: the occurrence of internal or external situations (single or in a combination of both), which result in an interruption of supplies of a medicinal product, if not properly addressed and controlled.
Common position paper (Charnay-Sonnek et al., 2013)	A situation in which the total supply of an authorized medicine or of a medicine used on a compassionate basis is inadequate to meet the current or projected demand at the patient level. The shortage may be local, national, European or international.
FIP (Fédération Internationale Pharmaceutique, 2013)	A medicine shortage can be defined as a drug supply issue requiring a change. It impacts patient care and requires the use of an alternative agent.
EAHC (Executive Agency for Health and Consumers, 2012)	The availability to patients of medicinal products in a pharmacy setting.
ASHP (Fox et al., 2009)	A supply issue that affects how the pharmacy prepares or dispenses a drug product or influences patient care when prescribers must use an alternative agent.
**Articles**
Costelloe et al., 2014	A drug shortage was defined as the inability to purchase a particular drug from wholesalers on a particular day.
Dragic, 2012	Drug shortage as every delay in monthly drug supply
Heiskanen et al., 2014	A drug supply issue requiring a change that impacts patient care and requires the use of an alternative agent.
Pauwels et al., 2015	A shortcoming in the supply of a medicinal product that affects the patient’s ability to access the required treatment in due time.

Seven definitions of drug shortages for reporting purposes were found on websites of (inter)national authorities; one more was obtained by contacting the Farmanco, the Dutch reporting database. Reporting websites are mostly nationally regulated, except for the database of EMA (European Medicines Agency, 2013; ANSM, 2014; Federaal Agentschap voor Geneesmiddelen en Gezondheidsproducten, 2014; Koninklijke Nederlandse Maatschappij ter bevordering der Pharmacie, 2014; Agencia Española de Medicamentos Y Productos Sanitarios - AEMPS, 2015; Australian Government - Department of Health - Therapeutic Goods Administration [TGA], 2015; BfArM - Lieferengpässe, 2015; Canadian Drug Shortage Database, 2015). **Table 2** displays an overview of these eight definitions.

**Table 2:** Overview of the reporting definitions of drug shortages.

Source	Definition
**National**
ANSM (ANSM, 2014)	Drugs for which unavailability cause a risk for public health and have no therapeutic alternative.
AEMPS (Agencia Española de Medicamentos Y Productos Sanitarios - AEMPS, 2015)	All drugs which experience supply problems are reported, except for those for which a rapid solution is expected.
BfArM (BfArM - Lieferengpässe, 2015)	A supply shortage is expected to go beyond 2 weeks interruption extradition to the usual extent or a significantly increased demand that cannot be adequately met. Only supply shortages of drugs listed, where special information needs of professionals are required. Currently, this is for prescription drugs that are intended primarily for the treatment of life-threatening or serious diseases for which no alternative preparations are available.
Belgian FAMHP (Federaal Agentschap voor Geneesmiddelen en Gezondheidsproducten, 2014)	Holders of the market authorization should notify the Belgian FAMHP when a drug will be unavailable for a time period longer than 14 days.
Farmanco (Koninklijke Nederlandse Maatschappij ter bevordering der Pharmacie, 2014)	All supply problems of drugs are reported if it is expected that the drug will be undeliverable for a time period longer than 14 days.
Canadian Drug Shortage Databank (Canadian Drug Shortage Database, 2015)	As soon as a market authorization holder knows that it will take longer than 20 days to supply a drug to meet expected patient volumes on an ongoing basis, they will report this as a shortage on the communications platform. It is understood that the inability of a patient to receive their prescribed medicines at the first attempt to fill a prescription may not constitute a drug being in ‘shortage,’ as the drug may be available in other pharmacies or within the wholesale or distribution network (i.e., pharmacy supply chain), usually within a few days.
Australian Medicine Shortages Information Initiative (Australian Government - Department of Health - Therapeutic Goods Administration [TGA], 2015)	This information is based on the voluntary notification by sponsors in accordance with the agreed Protocol.
**European**
EMA (European Medicines Agency, 2013)	Medicines shortages that affect or are likely to affect more than one EU MS, where the EMA has assessed the shortage and provided recommendations to patients and healthcare professionals across the EU.

Remarkable differences between the definitions, such as “when do supply problems become drug shortages”; “permanent or temporally discontinuation”; “typology” and “time frame”, will be discussed in further detail in the next subsections.

### When do Supply Problems become Drug Shortages?

Not every supply problem immediately causes a drug shortage. In the definitions, diverse wordings are found to describe when supply problems become drug shortages. Shortages can be expressed in four different ways, specifically according to (i) the demand side (e.g., when the supply of a drug is inadequate to meet the current or projected demand), (ii) the supply side (e.g., interruption of supply), (iii) the delivery of a drug (e.g., undeliverable), and (iv) the availability of drugs (e.g., the availability of drugs to patients). Additionally drug shortages can occur at two different levels, namely (a) at pharmacies or wholesalers and (b) at consumers’ level. The different expressions will be discussed in the next paragraphs.

#### Demand Side

With respect to the demand side, the formulation: “when the supply of a drug is inadequate to meet the current or projected demand” is found in five general definitions, including the definitions of the FDA, International Society Pharmaceutical Engineering (ISPE), Australian Health Department, Health Canada as well as the definition found in the common position paper by Charnay-Sonnek et al. (2013) (see **Table 1**) and one definition for reporting purposes, namely of the Canadian Drug Shortages Database (see **Table 2**; Food and Drug Administration, 2011b; Charnay-Sonnek et al., 2013; ISPE, 2013; Government of Canada - Health Canada, 2014; Australian Government - Department of Health - Therapeutic Goods Administration [TGA], 2015; Canadian Drug Shortage Database, 2015). These wordings indirectly suggest that supply problems at pharmacy level (i.e., an order that is not delivered) are not considered as drug shortages. As long as there is no demand for the drug which is experiencing supply problems, these organizations do not consider the situation as a drug shortage. The FDA, ISPE, and the Australian Health Department complemented these wordings with “at patient/user/consumer level” (Food and Drug Administration, 2011b; Charnay-Sonnek et al., 2013; ISPE, 2013; Australian Government - Department of Health - Therapeutic Goods Administration [TGA], 2015). A small difference should be acknowledged between “patient level” and “user or consumer level.” The latter includes patients as well as healthy people using the drug, e.g., in experiments.

#### Supply Side

Five definitions consider drug shortages at the level of the supply side using expressions as “inadequate supply,” “interruption of supply,” or “supply issue.” Three out of the five are general definitions, namely from the EFPIA, the Dutch Institute for Rational Use of Medicines, and the FIP (see **Table 1**) and additionally two reporting definitions: from the BfArM and AEMPS (see **Table 2**; Instituut voor Verantwoord Medicijngebruik, 2012; European Federation of Pharmaceutical Industries and Associations, 2013; Fédération Internationale Pharmaceutique, 2013; Agencia Española de Medicamentos Y Productos Sanitarios - AEMPS, 2015; BfArM - Lieferengpässe, 2015). These expressions do not indicate by whom, pharmacy or consumers, drug shortages should be perceived. Only the FIP indicates in the sequel of the definition that this supply issue impacts patient care (Fédération Internationale Pharmaceutique, 2013).

#### Delivery of a Drug

Definitions found in the Belgian and French laws, as well as the definition formulated by the EMA in 2014, use the phrase “undeliverable” as a basis for drug shortages (see **Table 1**; Wet Betreffende de Verplichte Verzekering Voor Geneeskundige Verzorging En Uitkeringen Gecoördineerd Op 14 Juli, 1994; Décret N° 2012-1096 Du 28 Septembre 2012 Relatif À L’approvisionnement En Médicaments À Usage Humain, 2015). Again a difference is noticed regarding the specified level: the Belgian law declares a situation as a drug shortage at the level of community pharmacies, hospital pharmacies or wholesalers, while the French law and EMA specify their definitions at patient level (Wet Betreffende de Verplichte Verzekering Voor Geneeskundige Verzorging En Uitkeringen Gecoördineerd Op 14 Juli, 1994; Décret N° 2012-1096 Du 28 Septembre 2012 Relatif À L’approvisionnement En Médicaments À Usage Humain, 2015).

#### Availability of a Drug

The AIFA and the EAHC define drug shortages as “the availability of drugs to patients” (see **Table 1**; Executive Agency for Health and Consumers, 2012; The Italien Medicines Agency, 2014). This expression clarifies at which level drug shortages are considered, however, it also regards access to medicines. The ANSM and the FAMHP use the expression of “unavailable” to specify the reporting conditions in France and Belgium (see **Table 2**; ANSM, 2014; Federaal Agentschap voor Geneesmiddelen en Gezondheidsproducten, 2014).

Considering the replies of the contacted organizations on the question: “when do supply problems become drug shortages,” different opinions were observed. Answers are very divergent and the following quotes highlight the differences between several organizations on their approach to evaluating supply problems.

“All (supply) interruptions should be evaluated for their potential to contribute to a shortage.” **[R1]**

“Supply problems should be considered as drug shortages when the pharmacist or medical doctor has to change the prescription.” **[R2]**

“A supply problem becomes a shortage as soon as it has consequences with regard to deliver medicines …” **[R3]**

### What about Permanent Discontinuation of Drugs?

Drug shortages can be temporally (e.g., quality problems) or permanent (e.g., withdrawal of the marketing authorization). As discussed above, diverse wording formulations are found to express drug shortages. Still it remains unclear for all of these expressions whether they include temporally as well as permanent discontinuation. After contacting different organizations, most organizations acknowledge the enormous consequences of permanent cessation of drugs, but do not consider it as a shortage, rather as an unavailability. The following quotes illustrate the different opinions:

“If a manufacturer decides to permanent discontinue a drug, there are probably alternative treatments on the market” **[R2]**

“The definition can imply the permanent discontinuation of a drug, because it means a shortage” **[R3]**

“The definition would include drugs that have been permanently discontinued until medical and pharmacy practice change in response to the discontinuation. Until prescribers stop prescribing a specific drug product, pharmacists will still be addressing the lack of availability, regardless of cause.” **[R4]**

### Time Frame

Three general definitions, namely from the Belgian and the French law and the Australian Health Department, incorporate a period during which the drug is unavailable. Only two of them indicate an exact time frame, both references are provided by national laws. The Belgian law declares “an uninterrupted period of four days” as time frame, while the French law defines drug shortages as situations where drugs are “undeliverable for three days” (Wet Betreffende de Verplichte Verzekering Voor Geneeskundige Verzorging En Uitkeringen Gecoördineerd Op 14 Juli, 1994; Décret N° 2012-1096 Du 28 Septembre 2012 Relatif À L’approvisionnement En Médicaments À Usage Humain, 2015). The Australian Health Department uses an undefined timeframe noting “not likely to meet the demand for a period of time” (Australian Government - Department of Health - Therapeutic Goods Administration [TGA], 2015). It remains unclear when the Australian government will consider supply problems as a shortage; this period can range from one day to one month or even longer.

Most organizations agree that a time frame in a definition is superfluous, since it occurs that for instance in the northern part of a country a shortage is experienced while in the southern part the drug is still available.

“In terms of a definition, then a time scale would seem to have little relevance.” [**R1**]

“… as soon as a pharmacist cannot deliver a prescription drug … there is a shortage.” [**R3**]

On the other hand, a specified time frame is often (in four of the eight definitions) used as a condition to report drug shortages (Federaal Agentschap voor Geneesmiddelen en Gezondheidsproducten, 2014; Government of Canada - Health Canada, 2014; Koninklijke Nederlandse Maatschappij ter bevordering der Pharmacie, 2014; BfArM - Lieferengpässe, 2015). Three databases will report shortages that go beyond 14 days, namely the FAMHP, Farmanco, and BfArM (Federaal Agentschap voor Geneesmiddelen en Gezondheidsproducten, 2014; Koninklijke Nederlandse Maatschappij ter bevordering der Pharmacie, 2014; BfArM - Lieferengpässe, 2015). The Canadian drug shortages database reports only drug shortages which will take longer than 20 days (Government of Canada - Health Canada, 2014). AEMPS uses an undefined time frame; they will report all shortages unless a rapid solution is expected (Agencia Española de Medicamentos Y Productos Sanitarios - AEMPS, 2015).

### Typology

Including a time frame is one way to decrease the workload of (inter)national authorities when reporting drug shortages. Another way to decrease the workload of reporting drug shortages is by making a typology of the affected disease classes. This approach is only observed in reporting definitions and thus should be considered as a condition to report shortages. Databases of Germany and France will only report drugs which are intended for the treatment of life-threatening or serious diseases for which no alternative preparations are available (ANSM, 2014; BfArM - Lieferengpässe, 2015). In Spain, Belgium, the Netherlands and Canada no explicit typology is included in the definition and thus all drugs experiencing supply problems will be reported (Federaal Agentschap voor Geneesmiddelen en Gezondheidsproducten, 2014; Koninklijke Nederlandse Maatschappij ter bevordering der Pharmacie, 2014; Government of Canada - Health Canada, 2014; Agencia Española de Medicamentos Y Productos Sanitarios - AEMPS, 2015).

## Discussion

This study provides an overview of 26 unique definitions for drug shortages as provided by two legislations, five governmental organizations, seven professional organizations, four from scientific articles, seven national reporting databases and one European reporting database (see **Tables 1** and **2**; Wet Betreffende de Verplichte Verzekering Voor Geneeskundige Verzorging En Uitkeringen Gecoördineerd Op 14 Juli, 1994; Fox et al., 2009; Food and Drug Administration, 2011b; Dragic, 2012; Executive Agency for Health and Consumers, 2012; Instituut voor Verantwoord Medicijngebruik, 2012; Charnay-Sonnek et al., 2013; European Medicines Agency, 2013; European Federation of Pharmaceutical Industries and Associations, 2013; Fédération Internationale Pharmaceutique, 2013; ANSM, 2014; Costelloe et al., 2014; Federaal Agentschap voor Geneesmiddelen en Gezondheidsproducten, 2014; Government of Canada - Health Canada, 2014; Heiskanen et al., 2014; ISPE, 2014; The Italien Medicines Agency, 2014; Agencia Española de Medicamentos Y Productos Sanitarios - AEMPS, 2015; Australian Government - Department of Health - Therapeutic Goods Administration [TGA], 2015; BfArM - Lieferengpässe, 2015; Canadian Drug Shortage Database, 2015; Décret N° 2012-1096 Du 28 Septembre 2012 Relatif À L’approvisionnement En Médicaments À Usage Humain, 2015; Pauwels et al., 2015). A summary of the different elements in definitions is presented, accompanied by the conditions which are currently used to report drug shortages. The differences and similarities were compared and quotation marks with underlying reasons for a better understanding of the definitions were added.

Currently two types of definitions are in circulation: general definitions to designate a drug shortage and reporting definitions to specify when to report a drug shortage. This distinction is also implemented in the results section due to essential differences between the two types of definitions. However, in the future, one should aim at aggregating both definitions into one to facilitate the comparison of reporting databases and scientific studies. A general definition should be developed to designate a drug shortage and additionally one must specify uniform conditions to report shortages. The general definition together with its reporting conditions should be developed in consultation with all stakeholders which are confronted with drug shortages and should be addressed to all different stakeholders. EU policy makers are best placed to set up a stakeholder forum where the main elements of the general definition together with its reporting definition can be discussed. We propose to include the definition and the reporting conditions in an EU Directive to reach more harmonized reporting databases based on the same definition, leaving the national authorities the responsibility on the means to achieve this goal.

To define this uniform definition for drug shortages, different decisions should be made. In the first place, one must determine which expression will be used to describe a drug shortage and at which level a drug shortage should be considered. At this moment four expressions are found: (i) when the supply cannot meet the demand, (ii) in case of an interruption of supply, (iii) when the drug is undeliverable, or (iv) when the drug is unavailable. Afterward the level of drug shortages should be determined. “At patient level” seems harder to identify in practice, while “an undelivered order” appears easier to detect. If the prices of drugs would be subjected to “free market” principles (i.e., a system in which prices for goods are set freely between vendors and consumers) a drug shortage, from an economic point of view, should be defined according to the demand side, because a shortage can only occur when the demand exceeds the supply (Simoens et al., 2005).

Whether to include permanent discontinuations is a third issue to be considered. Permanent discontinuations are subject to other rules in case of reporting the problem, therefore it might be easier to set proper conditions for temporary shortages and permanent ones (De Weerdt et al., 2015). Still it remains important to inform patients properly when a drug will be withdrawn from the market.

Incorporating a time frame in the general definition seems rather irrelevant for the following two reasons. First, only legislations contain time frames in their definitions. This can be explained by the necessity for the practical implementation of the legislations. Secondly, every country has its own policy on the delivery of drugs therefore it might be hard to come to one time frame which can be generally applied. However, including a time frame as a reporting condition should be considered a feasible method to reduce the workload of the competent authority, especially since four of the eight references already included a specified time frame (Federaal Agentschap voor Geneesmiddelen en Gezondheidsproducten, 2014; Koninklijke Nederlandse Maatschappij ter bevordering der Pharmacie, 2014; BfArM - Lieferengpässe, 2015; Canadian Drug Shortage Database, 2015). The three European references (Germany, The Netherlands, and Belgium) specified that a drug should be in shortage for at least 14 days (Federaal Agentschap voor Geneesmiddelen en Gezondheidsproducten, 2014; Koninklijke Nederlandse Maatschappij ter bevordering der Pharmacie, 2014; BfArM - Lieferengpässe, 2015), and perhaps this can be extended to all EU Member States’ databases.

Reducing the workload of competent authorities who are responsible for the drug shortages database can also be obtained by only including “drugs shortages which cause a risk for public health”. This is observed in Germany and France (ANSM, 2014; BfArM - Lieferengpässe, 2015). However, these restrictions will also misrepresent the size of the problem because not all shortages will be covered by the databases.

Differences between the found definitions of drug shortages can be partly explained by the diversity of stakeholders. Definitions in national legislations have other purposes than the definitions of drug shortages of professional organizations. This can be explained by the different roles, responsibilities and expectations of the different stakeholders. E.g. a time frame is found in national laws and not in definitions of professional organizations, probably because national legislations consider the specificities of the local drug market. However, this does not explain the differences within a group of the same stakeholders, which may be attributed to the different missions of the organizations. E.g., the definition of the EFPIA is very broad and will encounter a lot more drug shortages compared to the definition of the FIP. Although the differences between and within stakeholders, the fact that so many organizations are troubled with this problem and searching for solutions, harmonization on the definition of drug shortages at EU level seems possible.

A difference should be noted in the terms “drug shortage” and “drug unavailability”. A drug shortage is defined as “an interruption of supply chain”, while drug unavailability rather refers to “not introducing new, innovative drugs to the market” (De Weerdt et al., 2015). However, the Belgian federal agency denotes to *unavailability* instead of *shortage* and should be willing to reformulate its definitions in order to avoid confusion.

Obtaining a uniform definition for drug shortages is important as well as identifying which conditions are preferable to report drug shortages in order to facilitate international comparison. This paper can be used as a guidance to point out all the different elements which should be considered to formulate a uniform definition applied in the EU. Nevertheless, the definition should acknowledge different national legislations and opinions of diverse stakeholders. The only way to obtain this is to sit around the table with all stakeholders involved with supply problems, which should be the next step in this investigation.

## Author Contributions

All authors participated in the design of the study. EDW performed data collection and analysis and drafted the manuscript. IH, SS, MC revised the manuscript critically and contributed to the interpretation of the data. All authors read and approved the final manuscript.

## Conflict of Interest Statement

The authors declare that the research was conducted in the absence of any commercial or financial relationships that could be construed as a potential conflict of interest.

## Abbreviations

AEMPS: Spanish Agency of Medicines and Medical Devices (Spain)
AIFA: Italian Medicines Agency (Italy)
ANSM: French National Agency for Medicines and Health Products Safety (France)
ASHP: American Society of Hospital Pharmacies (US)
BfArM: Federal Institute for Drugs and Medical Devices (Germany)
EAHC: Executive Agency for Health and Consumers
EFPIA: European Federation of Pharmaceutical Industries and Associations
EMA: European Medicines Agency
FAMHP: Federal Agency of Medicines and Health Products (Belgium)
Farmanco: Royal Dutch Pharmacists Association Farmanco (the Netherlands)
FDA: Food and Drug Association (US)
FIP: International Pharmaceutical Federation
ISPE: International Society of Pharmaceutical Engineering
